# Determination of optimal PTV margin for patients receiving CBCT‐guided prostate IMRT: comparative analysis based on CBCT dose calculation with four different margins

**DOI:** 10.1120/jacmp.v16i6.5691

**Published:** 2015-11-08

**Authors:** Sukhdeep K. Gill, Krishna Reddy, Nina Campbell, Changhu Chen, David Pearson

**Affiliations:** ^1^ Department of Radiation Oncology University of Toledo Toledo OH USA; ^2^ Department of Physics University of Salford Salford UK

**Keywords:** CBCT, IMRT, planning CT, DVH

## Abstract

Variations in daily setup and rectum/bladder filling lead to uncertainties in the delivery of prostate IMRT. The purpose of this study is to determine the optimal PTV margin for CBCT‐guided prostate IMRT based on daily CBCT dose calculations using four different margins. Five patients diagnosed with low‐risk prostate cancer were treated with prostate IMRT to 70 Gy in 28 fractions using daily CBCT for image guidance. The prostate CTV and OARs were contoured on all CBCTs. IMRT plans were created using 1 mm, 3 mm, 5 mm, and 7 mm CTV to PTV expansions. For each delivered fraction, dose calculations were generated utilizing the pretreatment CBCT translational shifts performed and dosimetric analysis was performed. One hundred and forty total treatment fractions (CBCT sessions) were evaluated. The planned prostate CTV V100% was 100% for all PTV margins. Based on CBCT analysis, the actual cumulative CTVs V100% were 96.55%±2.94%,99.49%±1.36%,99.98%±0.26%, and 99.99%±0.05% for 1, 3, 5, and 7 mm uniform PTV margins, respectively. Delivered rectum and bladder doses were different as compared to expected planned doses, with the magnitude of differences increasing with PTV margin. Daily setup variation during prostate IMRT yields differences in the actual vs. expected doses received by the prostate CTV, rectum, and bladder. The magnitude of these differences is significantly affected by the PTV margin utilized. It was found that when daily CBCT was used for soft‐tissue alignment of the prostate, a 3 mm PTV margin allowed for CTV to be covered for 99% of cases.

PACS numbers: 87.55.dk‐, 87.57.Q‐

## I. INTRODUCTION

Prostate cancer is the most commonly diagnosed male cancer worldwide, excluding skin cancer.^(1)^ IMRT for prostate cancer can create a steep dose gradient between the target volume and the OARs, allowing for target dose escalation. This has simultaneously improved local control and reduced late toxicities.^(2)^

Because of the high dose gradient achieved in IMRT, it is important to utilize an adequate PTV margin to ensure CTV coverage. Image‐guided radiation therapy (IGRT) is used in the daily treatment of prostate cancer to assist in precise dose delivery and to maximize the sparing of normal structures. Two important considerations during prostate radiotherapy, addressed with daily IGRT, include pretreatment organ motion and bladder/rectum filling.

The use of cone‐beam computed tomography (CBCT) for IGRT allows for the reduction of daily setup errors. The traditional use of generous PTV margins during prostate IMRT may result in irradiation of unnecessarily large volumes of the rectum and bladder. Proper image guidance can reduce errors in radiotherapy delivery and allow for a reduction of the PTV margin, thereby achieving better dose conformation and possibly reducing rectal and bladder toxicity. However, variations in the daily setup and physiological changes in the volumes of the bladder and rectum can result in displacement and deformation of the prostate during treatment, consequently changing the dose distribution to the target and adjacent organs.^(3)^ Dosimetric studies have indicated that the DVH produced at the time of planning may not be an exact representation of the actual dose received during treatment.^(4)^ Translational shifts in the prostate during the course of radiotherapy have been reported. For instance, Meijer et al.^(5)^ reported shifts of 2 to 4 mm, and Juan‐Senabre et al.^(6)^ reported shifts of 7 to 9 mm, in the position of the prostate during a radiotherapy course.

The goal of this study was to determine the optimal PTV margin for adequate CTV coverage, while minimizing the dose received by the rectum and bladder during an entire radiotherapy course. Prior studies have determined PTV margins based on calculations using the van Herk formula;^(7)^ however, recent work has demonstrated the limitations of the van Herk formula for PTV margin calculation.^(8)^ A further criticism of these prior studies is that many were completed using EPID, instead of daily CBCT, for image guidance.

In this study, daily CBCT data was used to investigate the deviation in the delivered dose from the planned dose, for plans calculated with four different PTV margins. Although previous studies have investigated daily CBCT‐based dose calculations during prostate radiotherapy, our study is the first, to the best of our knowledge, that has rigorously evaluated four different PTV margins and utilized every CBCT acquired throughout the entire course of treatment (for each patient).

## II. MATERIALS AND METHODS

### A. Patients

This study was a retrospective analysis of five patients diagnosed with low‐ or intermediate‐risk prostate carcinoma (T1cN0M0). In these cases, the PTV did not include the full seminal vesicles.

### B. CT Simulation and Planning

**Figure 1:**
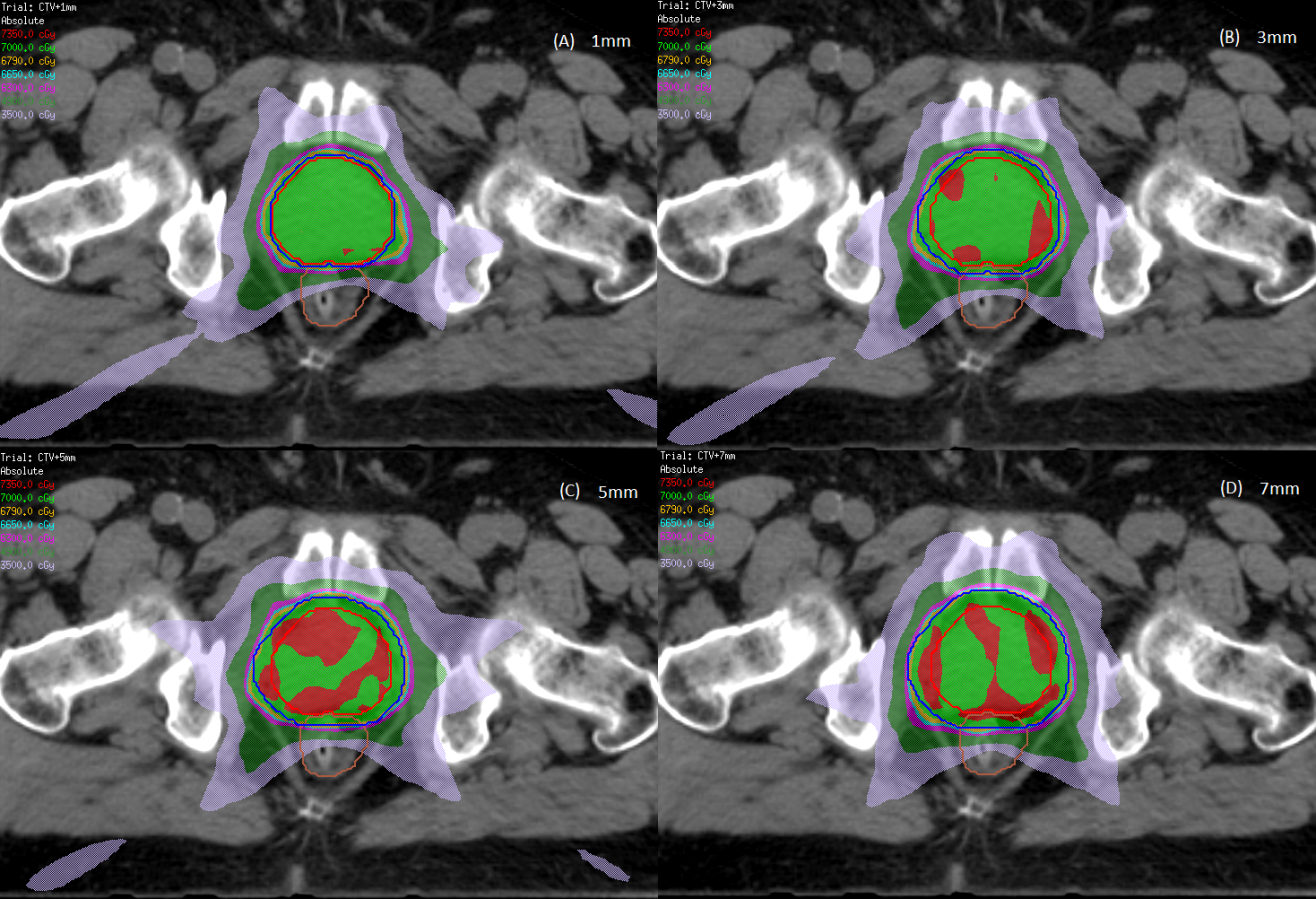
Comparison of the IMRT plan for 1, 3, 5, and 7 mm PTV margins on P‐CT.

**Figure 2:**
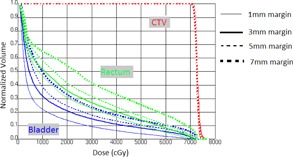
Comparison of CTV and OAR DVHs for 1, 3, 5, and 7 mm PTV margins on P‐CT.

Planning CT images were acquired using a Philips CT scanner (Philips Healthcare, Bothell, WA). Patients were scanned supine with a vacuum bag and knee wedge for immobilization. Three‐mm CT slices were acquired from the top of the iliac crest to 7 cm below the ischium. For each patient, four IMRT plans were generated using the Pinnacle^3^ treatment planning system (Koninklijke Philips, N.V., Amsterdam, The Netherlands). CTV was the contoured prostate. PTVs with 1, 3, 5, and 7 mm uniform margins from the CTV were created. All patients were planned using nine fields with gantry angles of 0° (AP), 40°, 80°, 120°, 160°, 200°, 240°, 280°, and 320°, using 10 MV photon beam IMRT. The prescribed dose was 70 Gy in 28 fractions (2.5 Gy per fraction). For all plans it was ensured that more than 95% of PTV was receiving the full prescription dose, and the CTV was covered by 100% of the prescription dose. For OARs, the following constraints were used: 1) rectum: V70<10%,V60<25%, and V70<10 cc;2)) bladder: V70<10% and V65<20%; 3) femoral heads: V50<5% and V35<15%. For consistency between plans on the same patient with differing margins, all plans were produced by the same physicist. IMRT planning for four different margins (1, 3, 5, and 7 mm) for one patient CT is shown in Fig 1. Additionally, plans were checked for consistency in the plan objectives. This involved ensuring that plans with larger margins resulted in higher rectum and bladder doses when compared to those on the same patient with tighter PTV margins. DVH for CTV, rectum, and bladder for four margins for one of the patients on CT used for treatment planning in Pinnacle is shown in Fig. 2. All plans were evaluated and approved by the treating physicians.

### C. CBCT acquisition

**Figure 3:**
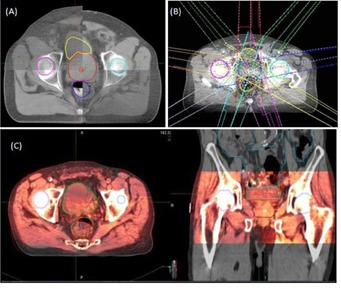
Soft tissue alignment (a) before treatment; (b) beam arrangement on daily CBCT; (c) rigid fusion of planning CT & daily CBCT to transfer contours.

All patients were treated on a TrueBeam linear accelerator (Varian Medical Systems, Palo Alto, CA). Prior to daily treatment, a kV‐CBCT of the pelvis was acquired using the standard "pelvis" mode settings: 125 kV, 80 mA, 13 ms, and full scan with half‐fan bowtie filter. An automatic match algorithm was used for the initial assessment of CBCT images and further verification was completed by a physician, with slight manual adjustments as deemed clinically necessary. Alignment was considered optimal when the posterior border of the prostate and the anterior surface of the rectum were in coincidence (Fig. 3).

### D. Contouring on CBCT

Daily CBCTs, including treatment isocenters, of all patients were exported from the "offline review" module in Varian's ARIA chart record system, to MIM Maestro software (MIM Software, Cleveland, OH). All contours and planned isocenters were transferred from the planning CT to the daily CBCT using image registration files, as shown in Fig. 3(c). The CTV was delineated on each CBCT. A rigid fusion between the planning CT and each CBCT was preformed using the translational shifts recorded in the image registration file of the patient's electronic chart. The CTV was then manipulated manually to take into account daily changes in the patient's anatomy that resulted in daily deformation of the prostate. To verify that these manual contour changes affected the prostate shape but not the volume, the volume of the CTV was recorded for each CBCT and compared to that of the planning CT. This method assumes that the prostate, on which the CTV is based, does not change in volume significantly from day to day. Additionally, the rectum, bladder, and femoral heads were contoured on each CBCT, according to RTOG guidelines.^(9)^

### E. Dose calculation on CBCT

CBCTs and structure files were transferred from MIM Maestro to Pinnacle^3^. Plans utilizing the different PTV margins were transferred from the planning CT to the daily CBCT treatment isocenter. The MU and weight for each beam was kept unchanged: the beam arrangement at the CBCT treatment isocenter is shown in Fig. 3(c).

**Figure 4:**
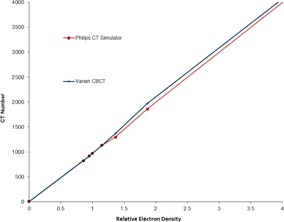
CT to Density calibration curve for Varian CBCT vs. Philips CTSIM.

For dose calculations, an anatomical site‐specific CT number to density calibration curve for CBCT calculation was used. The results of the CT number to density curve for the Philips Gemini CT and Varian OBI were in good agreement. A graphical comparison of CT to density curve for CT and daily CBCT is shown in Fig. 4. Raw data were exported from Pinnacle^3^ to a spreadsheet using script files.^(10)^ The optimal PTV margin was defined as that which would allow a minimum CTV coverage of 98% of the prescribed dose at least 95% of the time.

## III. RESULTS

Twenty‐eight daily CBCTs were used from each of the five patients who underwent image‐guided IMRT, resulting in a total of 140 CBCT datasets analyzed. The volumes of the prostate, rectum, and bladder on daily CBCTs as compared to the planning CTs are shown in Table 1.

**Table 1:** Prostate and OAR volumes

	*P‐CT*	*Daily CBCT*		
	*p‐CT Volume*	*Mean*	*Minimum*	*Maximum*
*Prostate Volume (cc)*				
Patient1	54.70	54.60	54.40	54.75
Patient2	59.45	59.80	59.40	61.00
Patient3	181.25	180.90	176.00	183.00
Patient4	92.81	92.10	90.54	92.81
Patient5	79.73	80.72	72.59	83.30
*Bladder Volume (cc)*				
Patient1	470.66	230.57	76.16	380.80
Patient2	69.89	91.30	64.49	133.06
Patient3	191.89	171.90	119.10	390.20
Patient4	75.36	128.61	50.66	373.51
Patient5	78.05	102.60	49.37	207.74
*Rectum Volume (cc)*				
Patient1	41.5	58.09	47.50	93.50
Patient2	83.4	92.17	69.66	109.69
Patient3	78.59	84.79	60.00	121.08
Patient4	76.59	84.26	68.84	110.19
Patient5	154.50	147.61	101.04	219.29

### A. CTV coverage

The CTV dose on the planning CT (P‐CT) compared to the cumulative result of all daily CBCTs (cum. CBCT) for 140 CBCT datasets is shown in Table 2. The CTV V100% calculated on the planning CT using 1, 3, 5, and 7 mm margins was 100%. For CBCTs, however, the cumulative CTV V100% was 96.55% for 1 mm, 99.49% for 3 mm, 99.98% for 5 mm, and 99.99% for 7 mm PTV margins. Similarly, the cum. CBCT CTV V95% was 99.33% for 1 mm, 99.90% for 3 mm, 100% for 5 mm, and 100% for 7 mm PTV margins.

**Figure 5:**
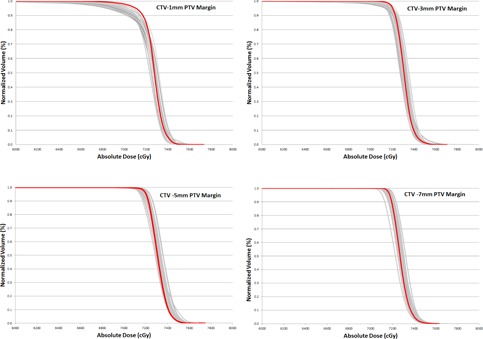
Comparison of the P‐CT (red) and daily CBCT (grey) CTV dose for one patient with margins of 1, 3, 5, and 7 mm.

For 1 mm PTV margins, the superior–inferior and anterior–posterior aspects of the prostate were not covered by 100% of the prescription dose. However, for 3–7 mm PTV margins, CTV coverage increased to >99%. The cum. CBCT CTV V95% was more than 99% for all four margins evaluated. The minimum cum. CBCT V100% was 78.87%, 90.43%, 97.50%, and 99.39% for 1, 3, 5, and 7 mm PTV margins, respectively (Fig. 5 shows the DVH).

The cumulative dose analysis for all CBCTs demonstrated a very meaningful increase in CTV coverage as the margin was increased from 1 mm to 3 mm, with a smaller increase in CTV coverage as the PTV margin was further incrementally increased beyond 3 mm.

**Table 2:** Comparison of CTV dose for P‐CT and cumulated daily CBCTs

	*1 mm PTV Margin (%)*		*3 mm PTV Margin (%)*	
	*Planned*	Cum.CBCT±SD	*Planned*	Cum.CBCT±SD
V107%	12.81	15.37±26.83	16.31	22.04±36.44
V100%	100.00	96.55±2.94	100.00	99.49±1.36
V98%	100.00	98.37±2.14	100.00	99.76±0.89
V95%	100.00	99.33±1.28	100.00	99.90±0.40
V90%	100.00	99.79±0.57	100.00	99.98±0.12
	*5 mm PTV Margin (%)*		*7 mm PTV Margin (%)*	
	*Planned*	Cum.CBCT±SD	*Planned*	Cum.CBCT±SD
V107%	20.85	27.16±38.86	25.06	29.02±37.84
V100%	100.00	99.98±0.26	100.00	99.99±0.05
V98%	100.00	100.00±0.15	100.00	100±0.00
V95%	100.00	100.00±0.08	100.00	100±0.00
V90%	100.00	100.00±0.00	100.00	100.00±0.00

### B. Bladder dose

Comparisons of the bladder dose for the planning CT vs. cum. CBCT for 1, 3, 5, and 7 mm PTV margins are shown in Table 3.

**Figure 6:**
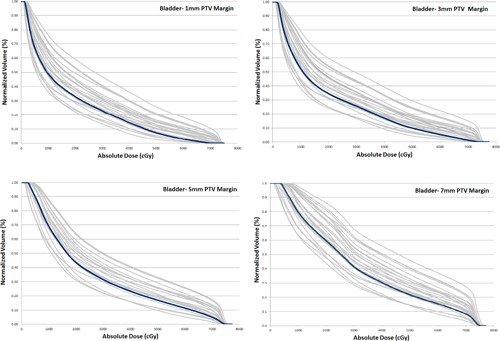
Comparison of the bladder dose for P‐CT (blue) and daily CBCTs (grey) for 1, 3, 5, and 7 mm PTV margins for one patient.

The mean planned V70 Gy was 3.49%, 5.61%, 9.56%, and 11.82% for 1, 3, 5, and 7 mm PTV margins, respectively. The V70 Gy (mean±SD) for cum. CBCT was 3.39%±5.38%,7.79%±9.59%,12.04%±12.50%, and 14.26%±13.16% for 1, 3, 5, and 7 mm PTV margins, respectively. As the PTV margin was increased, more variations in the bladder dose on the daily CBCTs versus the planning CT were observed (Fig. 6).

**Table 3:** Comparison of the planning CT and cumulative daily CBCT bladder dose

	*1 mm PTV Margin (%)*		*3 mm PTV Margin (%)*	
	*Planned*	Cum.CBCT±SD	*Planned*	Cum.CBCT±SD
V70 Gy	3.49	3.39±5.38	5.61	7.79±9.59
V65 Gy	6.78	5.25±7.18	9.70	11.43±11.71
V60 Gy	9.63	7.35±8.66	12.54	14.11±13.36
V50 Gy	14.86	11.47±10.88	17.38	19.16±16.16
V40 Gy	20.96	16.76±13.61	23.89	25.01±18.72
V30 Gy	27.82	22.60±17.91	31.75	32.00±21.97
	*5 mm PTV margin (%)*		*7 mm PTV margin (%)*	
	*Planned*	Cum.CBCT±SD	*Planned*	Cum.CBCT±SD
V70 Gy	9.56	12.04±12.50	11.82	14.26±13.16
V65 Gy	13.60	15.42±14.07	16.35	17.91±14.45
V60 Gy	16.11	17.99±15.32	18.98	20.64±15.43
V50 Gy	22.03	22.87±17.53	24.50	25.99±17.28
V40 Gy	26.93	28.25±19.52	30.58	32.05±18.96
V30 Gy	34.51	35.11±21.50	38.81	39.42±20.97

### C. Rectal dose

Comparisons of the rectal dose on the planned and cum. CBCT for 1, 3, 5, and 7 mm PTV margins are shown in Table 4.

**Figure 7:**
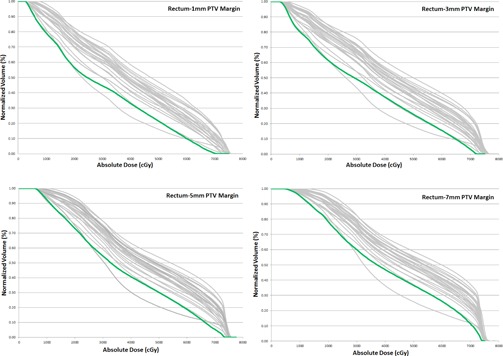
Comparison of the rectum dose for P‐CT (green) and daily CBCTs (grey) for 1, 3, 5, and 7 mm PTV margins for one patient.

Increasing the PTV margin led to considerable increases in rectal V70 (Fig. 7). The mean planned V70 Gy for 1, 3, 5, and 7 mm PTV margins was 0.97%, 4.37%, 7.86%, and 13.05%, respectively. The cum. CBCT V70 Gy (mean±SD) was 4.45%±3.84%,8.17%±4.98%,11.59%±5.69%, and 16.61%±6.79% for 1, 3, 5, and 7 mm PTV margins, respectively.

The 7 mm PTV margins did not reach the passing criteria for best PTV margins (i.e., V70<10%,V60<25%, and V70<10 cc;2)).

**Table 4:** Comparison of the P‐CT and cumulated daily CBCTs for rectum dose

	*1 mm PTV Margin (%)*		*3 mm PTV Margin (%)*	
	*Planned*	Cum.CBCT±SD	*Planned*	Cum.CBCT±SD
V70 Gy	0.97	4.45±3.84	4.37	8.17±4.98
V65 Gy	4.41	8.72±5.75	9.72	12.81±6.48
V60 Gy	8.08	12.35±7.18	14.17	16.53±7.49
V50 Gy	15.37	19.20±9.43	22.22	23.44±9.20
V40 Gy	23.18	26.41±11.62	30.5	33.66±10.96
V30 Gy	31.95	34.93±14.33	39.43	39.56±13.80
	*5 mm PTV Margin (%)*		*7 mm PTV Margin (%)*	
	*Planned*	Cum.CBCT±SD	*Planned*	Cum.CBCT±SD
V70 Gy	7.86	11.59±5.69	13.05	16.61±6.79
V65 Gy	13.63	16.59±7.10	19.82	21.90±8.21
V60 Gy	18.47	20.53±8.10	24.67	25.84±9.10
V50 Gy	26.83	27.58±9.66	32.85	32.90±10.48
V40 Gy	35.05	34.95±11.09	41.25	40.43±11.67
V30 Gy	44.7	44.34±14.00	51.04	49.97±13.82

Increasing the PTV margin led to considerable increases in rectal V70 (Fig. 7). The mean planned V70 Gy for 1, 3, 5, and 7 mm PTV margins was 0.97%, 4.37%, 7.86%, and 13.05%, respectively. The cum. CBCT V70 Gy (mean±SD) was 4.45%±3.84%,8.17%±4.98%,11.59%±5.69%, and %±6.79% for 1, 3, 5, and 7 mm PTV margins, respectively.

The 7 mm PTV margins did not reach the passing criteria for best PTV margins (i.e., V70<10%,V60<25%, and V70<10 cc;2)).

## IV. DISCUSSION

Daily setup variations and organ motion during prostate IMRT yield differences in the actual versus expected doses received by the prostate, rectum, and bladder. The magnitude of these differences is significantly affected by the PTV margins

Daily IGRT can improve the accuracy of treatment and reduce uncertainty. Several studies have suggested that PTV margins can be reduced with the use of IGRT. The majority of these have focused on translational shifts using EPID and PTV calculations based on the van Herk formula. Some of these studies and their findings are displayed in Table 5.

In the present study, the actual dose delivered to the prostate and OARs during an entire 28‐fraction course of radiation therapy was determined for each patient. Our work is unique in that each of the daily CBCT datasets gathered during the treatment course for five patients (a total of 140 CBCT datasets) was contoured, planned, and analyzed. The actual cumulative doses received by the prostate CTV, bladder, and rectum were compared to those determined based on the planning CT. For each CBCT dataset, four different dosimetric plans (utilizing PTV margins of 1, 3, 5, and 7 mm) were evaluated.

**Table 5:** CTV to PTV margin calculations for different studies using different image‐guidance strategies

		*CTV‐PTV Margins (mm)*			
*Reference*	*Method & Datasets*	*R‐L*	*A‐P*	*S‐I*	*Comments*
Current study	IMRT, daily CBCT n=140	3	3	3	CBCT dose calculation for entire treatment
Wu et al.^(16)^	3D CRT, n=148	3	3	3	16 CTs for each patient, bony alignment, van Herk formula for PTV calculation
Juan‐Senabre et al.^(6)^	IMRT, n=2884	7.3	9.0	7	Avg 27 CBCT per patient, van Herk formula used for PTV calculation
Skarsgard et al.^(15)^	3D CRT, n=736	3.6	3.7	3.7	EPID based corrections, van Herk formula used for PTV calculation
Meijer et al.^(5)^	IMRT, n=240	2	2	4	8 CT scans per patient, calculation based on 90% of the patients, minimal dose to CTV 95%
Cheung et al.^(17)^	IMRT, n=594	3	4	3	EPID before and after for first nine days, van Herk formula used for PTV calculation
van der Heide et al.^(18)^	IMRT, n=15,855	1.8	4.0	2.5	Daily EPID, van Herk formula used for PTV calculation

The received CTV doses differed from those calculated on the planning CTs during the planning process. Specifically, the mean cumulative CTV doses were lower than those determined on the planning CTs, especially with 1 mm PTV margins; CTV coverage increased with a PTV margin of 3 mm. Further increases in PTV margins (5 mm and 7 mm), yielded cumulative CTV coverage similar to that on the planning CTs. Cumulative CBCT dose analysis demonstrated a large increase in CTV coverage as the PTV margin was increased from 1 mm to 3 mm, but no significant increases in CTV coverage when the PTV margin was further increased to 7 mm. Increasing the PTV margin also increased the dose delivered to the bladder and rectum.

As the PTV margin was increased, more variations in the CBCT‐based bladder dose, compared to the planned dose, were observed. The actual cumulative doses received by the rectum were higher than the planned dose as the PTV margins were increased to 7 mm.

There are known issues associated with the use of CBCT for daily dose calculations.^(11)^ It has been suggested that dosimetric results from CBCT‐based dose calculations are comparable to those based on planning CTs. When using a site‐specific CT number for the density calibration of CBCT images, a 2% dose accuracy agreement has been observed between the planning CT and CBCT images in pelvic phantom studies.^(12)^ For dose calculations in this study, an anatomical site‐specific CT number to density calibration curve for CBCT calculation was used and our results between CT and daily CBCT are in a good agreement. This is shown in Fig. 4. It is important that HU numbers remain consistent over the time for the CBCT so that dose calculations to remain accurate. To confirm the stability of the CBCT an evaluation was performed using the monthly imaging tests of the Catphan 504 (Phantom Laboratory, Salem, NY). Analysis of these data showed minimal deviations in HU over a period of one year. Consistency of this agreement for the same CTSIM and daily CBCT has been shown previously as well.^(13)^

In future work, we will be evaluating changes in CTV and OAR doses for planning CT/daily CBCT alignment based primarily on soft tissue landmarks versus bony anatomy within the pelvis versus prostate/rectum interface. It is possible that the PTV margin necessary may be dependent on the type of registration used when aligning daily CBCTs. For instance, one study has reported that margins for bony anatomy alignment were larger compared to soft tissue alignment.^(14)^

## V. CONCLUSIONS

For prostate IMRT using daily CBCT for image guidance, a PTV margin of 1 mm is not sufficient to ensure acceptable prostate CTV coverage. A minimum PTV margin of 3 mm provides 100% CTV coverage for 99.49% of all treatments. Increasing the PTV margin to 5 mm achieved 100% CTV coverage for 99.98% of all CBCTs; however, the rectum V70 Gy almost doubled (compared to that received with a PTV margin of 3 mm). Considering both CTV coverage and OAR sparing, a uniform PTV margin of 3 mm, therefore, appears to be optimal for prostate radiotherapy.
